# Digital Pedoscopic Assessment and Data-Driven Classification of Pediatric Rearfoot Alignment

**DOI:** 10.3390/children12121633

**Published:** 2025-12-01

**Authors:** Yu-Sun Min

**Affiliations:** 1 Department of Rehabilitation Medicine, School of Medicine, Kyungpook National University, Daegu 41944, Republic of Korea; ssuni119@knu.ac.kr; 2 Department of Rehabilitation Medicine, Kyungpook National University Chilgok Hospital, Daegu 41404, Republic of Korea; 3 AI-Driven Convergence Software Education Research Program, Graduate School of Computer Science and Engineering, Kyungpook National University, Daegu 41566, Republic of Korea

**Keywords:** pediatric biomechanics, foot alignment, ankle pronation and supination, clustering analysis, pediatric rehabilitation

## Abstract

Background: Accurate assessment of pediatric foot biomechanics is challenging due to growth-related variability and limited quantitative tools. The supination and pronation angles of the ankle are critical for understanding lower limb alignment and pathological gait patterns. Objectives: This study introduces a novel digital pedoscopic system designed to enhance the quantitative evaluation of foot alignment and to demonstrate its clinical utility through clustering analysis of pediatric ankle angles. Methods: Thirty-five pediatric patients (mean age = 6.17 ± 4.54 years) with neurological or developmental disorders were evaluated using a semi-automated digital pedoscopic system to obtain quantitative measurements of ankle alignment. Key anatomical landmarks, including the heel, calf, and knee centers, were manually identified from posterior images, and the system automatically calculated ankle pronation and supination angles. K-means clustering analysis was applied to classify participants based on their biomechanical profiles. Results: A total of thirty-five pediatric patients were assessed, and the revised abstract now explicitly reports this sample size to improve clarity. Data-driven k-means clustering of bilateral rearfoot angles identified three clearly defined alignment subgroups—neutral, pronated, and supinated—each exhibiting characteristic distribution patterns and degrees of inter-individual variability. These findings highlight the system’s ability to quantitatively distinguish biomechanical phenotypes within a heterogeneous pediatric population. Visualization through scatter, box, and violin plots demonstrated distinct cluster-specific distributions and inter-individual variability in rearfoot alignment, demonstrating the feasibility of objective biomechanical stratification in pediatric populations. Conclusions: The digital pedoscopic imaging system provides a reliable and reproducible approach for quantitative assessment of foot alignment in children. Clustering analysis enables stratification of biomechanical subtypes, supporting individualized rehabilitation strategies and longitudinal monitoring in pediatric clinical practice.

## 1. Introduction

Accurate assessment of pediatric foot biomechanics is essential for diagnosing and managing lower-limb abnormalities, particularly in children with neurological or developmental disorders, where subtle gait deviations can have long-term functional consequences [1,2]. Conventional clinical methods—such as visual observation, manual goniometry, or footprint inspection—are inherently subjective and often lack quantitative precision and reproducibility [3,4,5]. This limitation is particularly relevant in pediatric populations, where rapid growth, developmental asymmetry, and neuromotor variability complicate reliable assessment [2,6,7].

Among the multiple kinematic parameters of the foot and ankle, the supination and pronation angles are key determinants of postural alignment and gait stability [8,9]. Deviations in these angles may result in pathological conditions such as genu valgum or varum, altered load distribution, and compensatory musculoskeletal strain [1,10,11]. Despite their clinical importance, only a few measurement systems allow for objective, repeatable quantification of these parameters in children, and existing three-dimensional gait laboratories are often resource-intensive and inaccessible in routine practice [12,13,14].

To overcome these gaps, a digital image-based framework was established for standardized evaluation of pediatric foot alignment. The system combines mirror-based plantar imaging with synchronized posterior-view acquisition, allowing concurrent visualization of plantar load distribution and coronal-plane alignment. Automated identification of anatomical landmarks at the malleoli and knees enables quantitative calculation of ankle pronation and supination angles, reducing examiner dependence and enhancing reproducibility. In addition to measurement accuracy, the framework integrates a data-driven clustering approach that stratifies pediatric patients according to biomechanical profiles and facilitates the identification of subgroups with distinct postural or neuromuscular characteristics [15,16,17,18]. Recent gait research has highlighted the potential of such data-driven subgrouping to reveal latent motor-control phenotypes across heterogeneous clinical populations [8,17,19].

Previous research has shown that children with sensory integration or neurological disorders frequently present limited ankle mobility and abnormal alignment patterns, highlighting the necessity for quantitative monitoring systems [20,21,22]. Building on these findings, the present study applies clustering-based analysis of quantitative ankle parameters to explore its potential for objective classification of pediatric alignment profiles in clinical rehabilitation contexts.

## 2. Materials and Methods

### 2.1. Participants

Thirty-five pediatric patients (21 females, 14 males; mean age = 6.17 ± 4.54 years) who visited the Department of Rehabilitation Medicine for gait or lower-limb alignment evaluation were enrolled. Participants presented with diverse clinical indications, including developmental delay, neurological conditions, and lower-limb malalignment, representing a broad spectrum of functional impairments typically encountered in pediatric rehabilitation practice. The study was conducted in accordance with the Declaration of Helsinki and approved by the Institutional Review Board of Kyungpook National University Chilgok Hospital (IRB No. KNUCH 2023-09-031). Written informed consent was obtained from the parents or legal guardians of all participants prior to data collection.

Eligible participants were children aged 1 to 18 years who were referred for evaluation of gait abnormalities, foot alignment concerns, or suspected neuromotor dysfunction and were able to stand independently on the pedoscopic platform for image acquisition. Children with neurological, neuromuscular, developmental, genetic, or musculoskeletal conditions potentially affecting foot alignment were included (Table A1). Participants were excluded if they were unable to stand safely due to severe motor impairment or behavioral difficulties, had an acute lower-limb injury or recent orthopedic surgery that could interfere with weight-bearing posture, or if inadequate posterior images prevented accurate anatomical landmark identification and RCSP angle computation.

### 2.2. Digital Pedoscopic Imaging and Angle Computation

**Figure 1:**
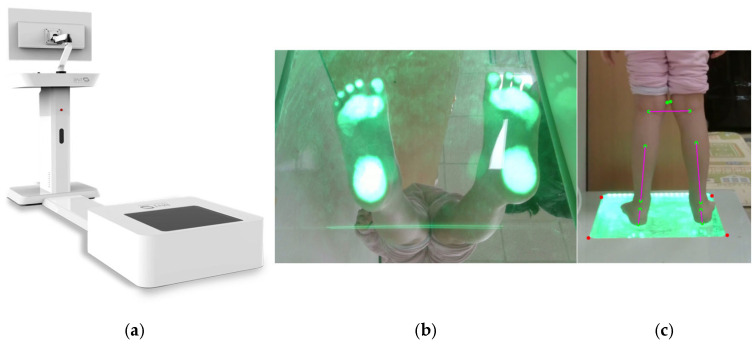
Digital pedoscopic imaging framework for quantitative assessment of pediatric foot biomechanics. (**a**) the mirror-integrated imaging system captures simultaneous plantar and posterior views of both lower limbs for alignment analysis; (**b**) the plantar view provides high-resolution visualization of the foot sole, illustrating plantar contact and load distribution; and (**c**) the posterior coronal-plane view enables calculation of rearfoot pronation and supination angles, facilitating simplified screening of foot deformities and automated generation of quantitative assessment reports. Green dots indicate manually identified anatomical landmarks, and red-dotted boxes delineate the foot-plate reference frame used to compute the plantar surface orientation.

A mirror-based digital pedoscopic imaging system (BNT Korea Co., Ltd., Daegu, Republic of Korea) was used to obtain high-resolution plantar and posterior images of both lower limbs. The system consists of a mirror-integrated platform and two synchronized cameras that simultaneously capture the plantar surface and coronal-plane view, enabling comprehensive evaluation of lower-limb alignment. Children were instructed to stand barefoot on the pedoscopic platform in a relaxed, natural stance with both feet placed shoulder-width apart and the arms resting comfortably at their sides. Participants were asked to look straight ahead at a fixed visual target positioned at eye level to minimize head or trunk deviation. Only one trial per participant was required because the digital pedoscopic system captures a synchronized posterior and plantar image instantaneously (within <1 s). If the child exhibited motion, imbalance, or altered posture during the capture, the trial was repeated. No participant required more than two attempts. The plantar view provides a detailed image of the foot sole, allowing visualization of medial–lateral load distribution and arch morphology, whereas the posterior view captures the coronal-plane alignment of both lower limbs from the heel to the knee. Key anatomical landmarks—including the heel bottom and center points, and the mid-calf and bilateral knee centers—were manually identified by the examiner within the posterior images. The resting calcaneal stance position (RCSP) line was defined by the vector connecting the heel bottom and heel center, representing the rearfoot axis used to quantify varus–valgus alignment. The tibia line was defined by the vector connecting the mid-calf and heel center, representing shank alignment relative to the rearfoot. The knee position angle was defined as the deviation of the tibial shaft from the vertical reference line passing through the heel bottom, representing coronal-plane knee alignment. Based on these user-defined landmarks, the system performed semi-automated calculations of ankle alignment angles corresponding to pronation and supination. Positive values indicated rearfoot pronation (valgus), negative values indicated rearfoot supination (varus), and 0° denoted neutral alignment. Before each recording session, a standardized calibration procedure was performed to minimize optical distortion and ensure geometric consistency across all measurements, thereby enhancing the reliability and reproducibility of the data (Figure 1).

### 2.3. Data Analysis

#### 2.3.1. Primary Variable and Sign Convention

The primary outcome variable was the signed rearfoot angle, derived from the resting calcaneal stance position (RCSP, degrees). For each limb, the coronal-plane ankle angle was calculated from the vector connecting the calcaneal midline and the tibial (knee) center identified in the posterior image. Positive values represented rearfoot pronation (valgus), negative values indicated rearfoot supination (varus), and 0° denoted neutral alignment. To maintain consistent directionality across participants, image coordinates were standardized for left and right limbs and verified through calibration procedures.

#### 2.3.2. Descriptive Statistics and Asymmetry

**Figure 2:**
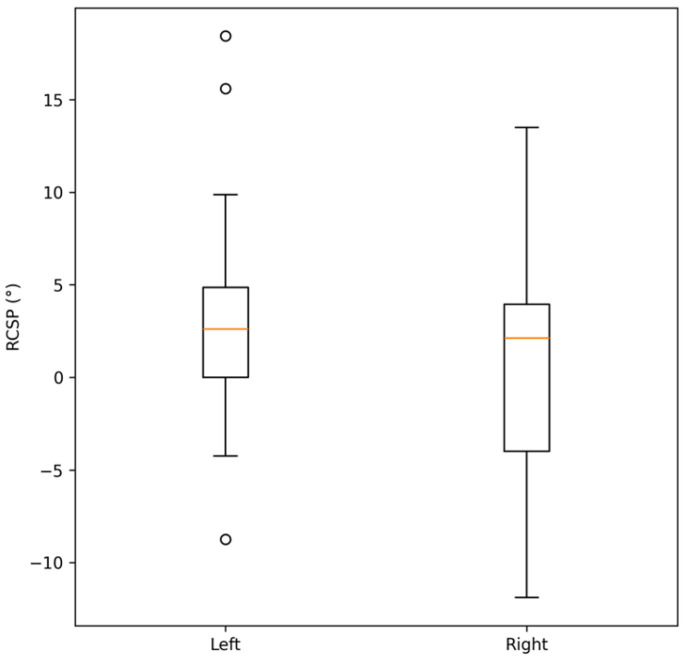
Box plot illustrating the distribution of signed RCSP (Rearfoot Center of Pressure) angles for the left and right ankles in pediatric patients. Positive values indicate pronation, negative values indicate supination, and zero represents a neutral alignment. The median left RCSP was slightly higher than the right, with both showing a wide range and presence of outliers. This plot highlights the inter-individual variability and asymmetry in rearfoot alignment.

For each ankle, the median, interquartile range (IQR), and full range of RCSP angles were reported, and their distributions were visualized using box plots. Inter-individual variability and left–right asymmetry were illustrated with a two-dimensional scatter plot of (Right RCSP, Left RCSP), where dashed reference lines at 0° represented neutral alignment (Figure 2). Within-participant asymmetry was calculated as the absolute difference between sides (|Right − Left|, degrees) and summarized descriptively in the text. While descriptive statistics provide an overview of group-level tendencies, they fail to capture the biomechanical diversity observed at the individual level. This limitation underscores the need for unsupervised clustering to stratify patients based on distinct alignment profiles rather than aggregated central values.

#### 2.3.3. Clustering and Model Validation

To address this heterogeneity, k-means clustering was applied to a two-dimensional feature space

$$x=\left[x_{1},x_{2}\right]=\left[\mathrm{Right}\;\mathrm{RCSP},\mathrm{Left}\;\mathrm{RCSP}\right].$$

Features were standardized to zero mean and unit variance before clustering. The k-means algorithm was initialized using the k-means++ method and executed with 20 random initializations; the solution minimizing the within-cluster sum of squares (inertia) was retained. Candidate models with k=2 to 6 were evaluated using: (i) elbow analysis of inertia vs. k, and (ii) mean silhouette coefficient based on Euclidean distances. To assess clustering stability, adjusted Rand indices (ARI) were computed across random initializations.

#### 2.3.4. Statistical Comparisons

Between-cluster statistical comparisons were performed using the Kruskal–Wallis test, followed by post hoc Dunn–Bonferroni corrections. The resulting *p*-values were accompanied by corresponding effect sizes (η^2^ for Kruskal–Wallis tests and rank-biserial correlations for pairwise contrasts). Left–right differences within clusters were examined using the Wilcoxon signed-rank test to assess asymmetry. All analyses were conducted using Python (version 3.10; Python Software Foundation, Wilmington, DE, USA). Numerical and data processing were performed with NumPy (v1.24) and pandas (v1.5), while clustering and validation procedures (k-means, silhouette analysis) were implemented using scikit-learn (v1.3). Visualization and figure generation were carried out with Matplotlib (v3.8). A fixed random seed (2025) was applied across all analyses to ensure reproducibility. The processing code and minimal anonymized RCSP datasets are available from the corresponding author upon reasonable request.

## 3. Results

A total of thirty-five pediatric participants were included in the final analysis. Anatomical landmarks were reliably extracted for all participants, resulting in valid and complete rearfoot alignment (RCSP) measurements for both ankles.

### 3.1. Distribution of Rearfoot Angles

The signed RCSP values exhibited substantial inter-individual variability and moderate left–right asymmetry (Figure 2). Positive angles corresponded to rearfoot pronation, negative angles to supination, and 0° indicated neutral alignment. The median RCSP angle on the left side was slightly greater than that on the right, suggesting a mild pronation tendency in the left ankle. Several outliers were observed in both limbs, indicating heterogeneous rearfoot alignment patterns within the cohort.

### 3.2. Cluster Identification and Characteristics

**Figure 3:**
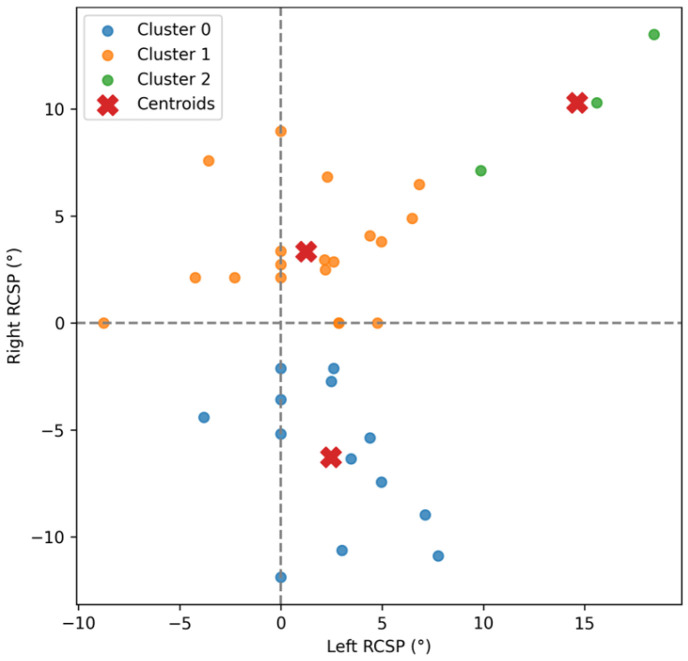
Scatter plot of signed rearfoot angles (RCSP) for the left and right ankles in all participants, color-coded by k-means cluster membership (k = 3). Cluster 0 (blue) represents participants with RCSP values near zero, indicating near-neutral rearfoot alignment. Cluster 1 (orange) includes individuals with higher positive RCSP angles, reflecting moderate to marked pronation. Cluster 2 (green) comprises participants with negative RCSP angles, corresponding to rearfoot supination or varus alignment. Each dot corresponds to a participant. Red “X” markers indicate the centroids of each cluster. Dashed lines at zero denote the neutral alignment threshold for both ankles, visualizing the distribution and asymmetry of rearfoot alignment profiles among pediatric patients.

K-means clustering of the two-dimensional feature space $x=\left[x_{1},x_{2}\right]=\left[\mathrm{Right}\;\mathrm{RCSP},\mathrm{Left}\;\mathrm{RCSP}\right]$ revealed three distinct biomechanical subgroups (Figure 3).

- Cluster 0 (blue): Participants with RCSP values close to zero, representing near-neutral rearfoot alignment.
- Cluster 1 (orange): Individuals with higher positive RCSP angles, indicative of moderate to marked rearfoot pronation.
- Cluster 2 (green): Participants with negative RCSP angles, reflecting rearfoot supination or varus alignment.

**Figure 4:**
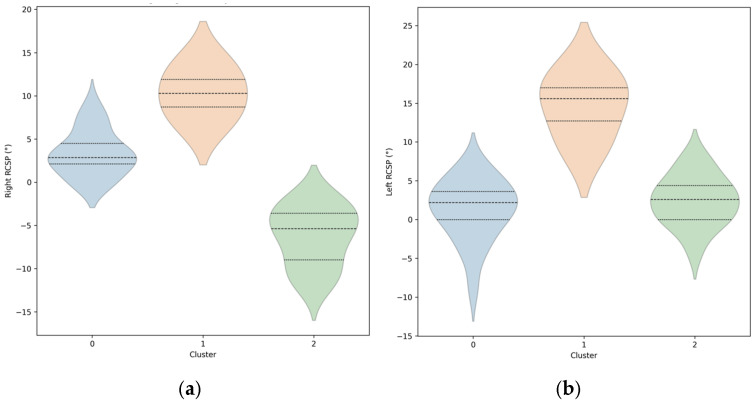
Violin plots illustrating the distribution of rearfoot center of standing posture (RCSP) angles for the right and left ankles across three biomechanical clusters derived from k-means analysis. Cluster 0 (blue) represents participants with near-neutral rearfoot alignment, Cluster 1 (orange) corresponds to individuals with higher positive RCSP values indicating moderate to marked pronation, and Cluster 2 (green) includes participants with negative RCSP values reflecting rearfoot supination or varus alignment. Each violin plot represents the probability density of RCSP values within each cluster, with horizontal dashed lines indicating the mean and median. Together, these plots illustrate distinct, cluster-specific distributions of rearfoot alignment patterns in the pediatric cohort. (**a**) RCSP distributions for right ankles across the three clusters.; (**b**) RCSP distributions for left ankles across the three clusters.

These clusters captured clear differences in both the magnitude and symmetry of rearfoot alignment, as further visualized in the violin plots (Figure 4). Cluster 1 (orange) exhibited the highest and most consistent RCSP values on both sides, indicating marked rearfoot pronation with relatively low variability. Cluster 0 (blue) demonstrated a narrower distribution centered near mild pronation, suggesting near-neutral alignment with moderate spread. Cluster 2 (green) showed the widest spread and most negative RCSP angles, reflecting substantial inter-individual variability in supinated or varus alignment profiles (Table 1).

### 3.3. Model Validation

**Figure A1:**
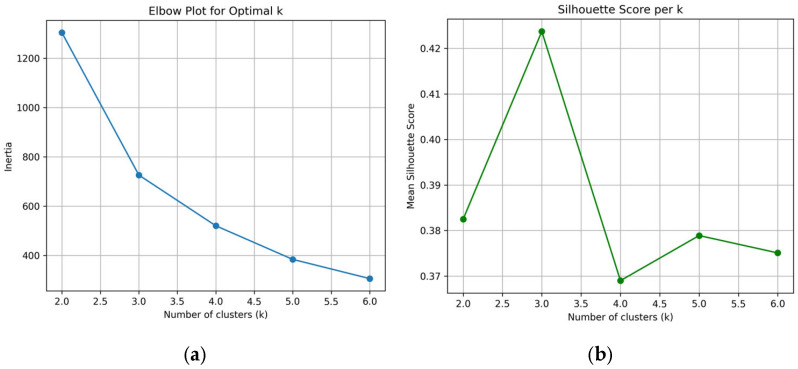
Clustering model selection results using two validation approaches: (**a**) Elbow plot illustrating the within-cluster sum of squares (inertia) for k-means clustering with k ranging from 2 to 6. A distinct inflection point is observed at k = 3, suggesting this configuration offers an optimal trade-off between model simplicity and explanatory power; (**b**) Silhouette analysis showing the mean silhouette scores for the same range of cluster numbers. The score peaked at k = 3, indicating that the three-cluster solution yields the best balance of intra-cluster cohesion and inter-cluster separation. Together, these results support the choice of k = 3 for stratifying pediatric patients based on rearfoot alignment profiles.

The optimal number of clusters was determined as k=3 based on two complementary criteria (Figure A1). The elbow plot demonstrated a distinct inflection at k=3, representing an optimal trade-off between model simplicity and explanatory power. Similarly, silhouette analysis achieved its highest mean score at k=3, indicating well-separated and internally coherent clusters. To assess clustering stability, adjusted Rand indices (ARI) were computed across random initializations. A median ARI greater than 0.90 confirmed the reproducibility and robustness of the clustering configuration.

### 3.4. Clinical Interpretation

The three clusters identified through k-means analysis represent distinct and clinically meaningful rearfoot alignment profiles in the pediatric population. Cluster 0 (blue) comprised participants with RCSP values near zero, corresponding to near-neutral rearfoot posture. Clinically, this group likely reflects children with typical biomechanical alignment who do not require specific intervention beyond routine observation. Cluster 1 (orange) included individuals with higher positive RCSP angles, indicating moderate to marked rearfoot pronation. This alignment pattern may be associated with ligamentous laxity, flatfoot posture, or muscular imbalance. Children in this cluster may benefit from interventions such as orthotic support, proprioceptive training, or targeted strengthening of the foot and ankle musculature to improve alignment and prevent functional instability. In contrast, Cluster 2 (green) consisted of participants with negative RCSP angles, reflecting rearfoot supination or varus alignment. This profile may be indicative of restricted ankle eversion or altered neuromuscular control, which is often observed in children with neurological or musculoskeletal conditions. Management strategies for this group could include stretching programs, neuromotor re-education, or further orthopedic evaluation, particularly in cases presenting with gait deviations or asymmetry. Overall, these findings highlight the clinical utility of digital pedoscopic imaging combined with clustering analysis in objectively classifying pediatric foot alignment and guiding individualized assessment and intervention strategies.

## 4. Discussion

This study demonstrated that quantitative assessment of pediatric rearfoot alignment using a digital pedoscopic imaging system, combined with data-driven clustering analysis, enables objective stratification of biomechanical subtypes among children with heterogeneous developmental and neurological profiles. The findings highlight the feasibility of integrating simple image-based measurements—specifically the Resting Calcaneal Stance Position (RCSP) angles—into clinical screening workflows for pediatric gait and alignment assessment. The identified clusters demonstrate meaningful biomechanical differences that align with known principles of rearfoot function.

### 4.1. Rearfoot Alignment and Its Role in Gait and Posture

Rearfoot pronation and supination are key determinants of lower-limb kinematics and load distribution during standing and walking [8,21]. Excessive pronation, for instance, alters medial arch mechanics and may induce internal tibial rotation and knee valgus, potentially affecting postural stability and gait efficiency [23,24]. Previous studies have documented that abnormal rearfoot alignment can influence plantar pressure and spatiotemporal gait parameters in both healthy and pathological populations [25,26]. For example, Fujishita et al. reported that adolescent athletes with rearfoot eversion exhibited greater forefoot loading and altered ankle motion during walking, emphasizing the biomechanical significance of rearfoot alignment in functional mobility [26]. Similarly, the comprehensive review by Jiang et al. underscored that foot morphology and alignment in children vary widely with age, growth, and sex, often manifesting as pronated or supinated foot postures during early development [2]. These observations align with the current study, where Cluster 2 (negative RCSP angles) represented children with rearfoot supination or varus alignment. This profile was also associated with greater left–right asymmetry, suggesting a potential link to early compensatory postural strategies or underlying neuromuscular constraints. Clinically, excessive supination may reflect limited ankle eversion, altered weight distribution, or rigidity in the subtalar joint [27,28,29]. Such patterns are often observed in children with atypical motor development, including those with cerebral palsy, sensory processing differences, or idiopathic toe-walking tendencies [22,30]. Furthermore, persistent supinated alignment may increase the risk of lateral instability or inefficient force transmission during gait, warranting early identification and targeted intervention [27]. These findings underscore the importance of quantifying rearfoot posture in both pronation and supination directions, as both extremes may have meaningful implications for musculoskeletal development and long-term functional outcomes in pediatric populations [2,31,32].

Although RCSP is a static measure, prior studies indicate that rearfoot alignment is meaningfully related to dynamic gait behavior. Increased pronation in standing has been associated with greater tibial internal rotation, altered knee loading, and characteristic plantar-pressure and spatiotemporal patterns during walking [23,24,26]. Static foot posture indices have also been shown to correlate with dynamic foot–ankle kinematics in children [33], although some evidence suggests that static alignment does not fully predict dynamic function [34]. These findings provide biomechanical context for the current RCSP-based clusters and support their potential relevance to dynamic gait characteristics.

### 4.2. Value of Clustering Analysis in Pediatric Biomechanics

The application of k-means clustering to RCSP data revealed three biomechanical subgroups—restricted, neutral, and pronated profiles—highlighting the potential of unsupervised learning for data-driven stratification in pediatric rehabilitation [8,15]. Recent work supports this approach: Vandekerckhove et al. used gait-based clustering to classify subgroups of children with Duchenne muscular dystrophy and identified distinct motor control patterns across disease stages [15]. Likewise, Mousavi et al. demonstrated that gait retraining targeting excessive pronation can effectively modify kinematic and kinetic parameters, underscoring the clinical value of categorizing patients by biomechanical subtypes [8]. The present findings extend these insights to a pediatric population using a simplified imaging-based approach rather than full 3D motion capture, making it accessible and practical in routine clinical settings.

### 4.3. Clinical Implications

The three clusters identified in this study represent clinically meaningful subtypes of rearfoot alignment in the pediatric population. Cluster 0 (blue) comprised participants with RCSP values near zero, indicative of near-neutral rearfoot posture. Clinically, this group likely reflects children with typical biomechanical alignment who may only require periodic monitoring for developmental changes in alignment. Cluster 1 (orange) included individuals with higher positive RCSP angles, corresponding to moderate to marked pronation. Such alignment patterns may reflect underlying ligamentous laxity, flexible flatfoot, or muscular imbalance. Children in this group may benefit from targeted interventions such as orthotic support, proprioceptive training, or strengthening of intrinsic and extrinsic foot muscles to improve stability and reduce the risk of functional deterioration. Cluster 2 (green) consisted of participants with negative RCSP angles, reflecting supination or varus rearfoot alignment. This profile may be associated with restricted ankle eversion or atypical neuromuscular control, commonly observed in children with neurological conditions or rigid foot deformities. Management strategies for this group may include stretching programs, neuromotor re-education, and referral for orthopedic assessment, particularly when asymmetry or gait deviations are present. Collectively, these findings underscore the clinical utility of combining digital pedoscopic imaging with clustering analysis to enable objective classification of pediatric rearfoot alignment and to support the development of individualized rehabilitation or monitoring strategies.

In Cluster 1, children exhibited moderate to marked rearfoot pronation, a pattern that corresponds to flexible flatfoot profiles frequently encountered in pediatric practice [8,35]. or such alignment, non-surgical strategies such as foot orthoses and structured exercise programs have been shown to improve pain, radiographic alignment, and balance in children with flexible pes planus or excessive pronation [8,23,36,37,38,39]; therefore, orthotic support combined with intrinsic foot muscle strengthening and neuromuscular or balance training may be particularly appropriate for symptomatic cases in this cluster, whereas asymptomatic children may simply be monitored over time [35,36]. In contrast, Cluster 2 included children with supinated or varus rearfoot alignment, which resembles pediatric pes cavus or cavovarus presentations [40]. Contemporary reviews emphasize that customized foot orthoses, ankle–foot orthoses when necessary, footwear modification, and balance or proprioceptive training can improve stability, reduce pain, and mitigate progression of deformity in cavus-type feet [40,41,42]. Accordingly, our suggestions for targeted stretching, neuromotor re-education, and orthotic evaluation in this cluster are consistent with these evidence-based recommendations [40,41,42]. By comparison, Cluster 0 consisted of children with near-neutral rearfoot alignment and minimal deviation from 0°. For this group, our recommendation of periodic observation without active intervention aligns with pediatric foot-care literature, which generally discourages treatment in asymptomatic children with physiologic or near-neutral foot posture unless pain, functional limitations, or progressive deformity are observed [35,36].

These interpretations are consistent with Molina-García et al. who showed that foot type (pronated, neutral, or supinated) was significantly associated with muscle strength, joint laxity, and plantar pressure parameters in children aged 5–10 years [3]. Moreover, Liu et al. found that typically developing children exhibit measurable left–right asymmetries in plantar pressure during walking and turning, supporting the interpretation that inter-limb asymmetry detected in our RCSP data reflects genuine biomechanical variability [43]. From a clinical standpoint, such stratification could guide early screening and personalized rehabilitation planning—particularly for children with flexible flatfoot, neuromotor delay, or asymmetric posture—using minimal equipment and short acquisition time.

### 4.4. Limitations and Future Directions

Several limitations should be acknowledged. First, the sample size was modest, and subgroup analyses for specific neurological or developmental diagnoses were not feasible. Second, the measurements were obtained under static or quasi-static standing conditions; dynamic gait cycles were not captured. Prior research has shown that static alignment does not always correspond directly to dynamic function [2,33,34]. Third, the current clustering model used only bilateral RCSP angles as input features. Integrating multi-modal data—such as plantar pressure distribution, 3D joint kinematics, or electromyographic patterns—may further enhance classification robustness. Fourth, because repeated image marking was not performed within the present dataset, formal intra- and inter-rater reliability metrics for landmark identification could not be calculated. This represents a methodological limitation and will be addressed in a prospective follow-up study designed to evaluate measurement reproducibility for digital pedoscopic RCSP assessment. Fifth, the digital pedoscopic system used in this study required manual identification of anatomical landmarks on posterior images. Because this step relies on examiner input, subtle differences in landmark placement may influence the calculated RCSP angles and could affect the reproducibility of the measurements. Although previous studies have noted similar challenges in image-based foot alignment assessment, future implementations should aim to reduce rater-dependent variation by incorporating automated landmark-detection algorithms, computer-vision–assisted calibration, or standardized examiner training protocols. Finally, the present findings were derived from a clinically heterogeneous sample consisting of children with neurological, developmental, genetic, and musculoskeletal conditions. As such, the alignment profiles and cluster characteristics identified in this study may not be directly generalizable to typically developing children with physiologic foot posture. Future research should expand the dataset to include larger and more diverse pediatric cohorts, validate the identified clusters longitudinally, and explore whether targeted interventions (e.g., orthotic correction, gait retraining, neuromuscular re-education) can shift patients between biomechanical clusters over time.

## 5. Conclusions

This study shows that digital pedoscopic imaging combined with clustering analysis can objectively classify rearfoot alignment patterns in pediatric patients. Although manual landmark identification remains the main limitation, the framework provides a practical and reproducible approach for evaluating pronated, neutral, and supinated alignment profiles in clinical settings. These stratified patterns may support early screening and individualized rehabilitation planning. Future work should focus on automating landmark detection, including typically developing children to improve generalizability, and integrating dynamic gait data to further strengthen clinical utility.

## Figures and Tables

**Table 1:** Descriptive statistics (Mean ± SD) of RCSP angles and left–right asymmetry across clusters. Positive RCSP values indicate pronation, whereas negative values indicate supination.

Cluster	Rt RCSP (Mean ± SD)	Lt RCSP (Mean ± SD)	Left-Right Asymmetry (Mean ± SD)
0	3.34 ± 2.64	1.24 ± 3.92	3.56 ± 3.24
1	10.31 ± 3.19	14.63 ± 4.36	4.32 ± 1.38
2	−6.28 ± 3.42	2.46 ± 3.23	8.74 ± 5.63

## Data Availability

The minimal anonymized dataset and analysis code are publicly available on Zenodo at https://doi.org/10.5281/zenodo.17595442.

## Abbreviations

The following abbreviations are used in this manuscript:

RCSP	Resting Calcaneal Stance Position
IQR	Interquartile Range
IMU	Inertial Measurement Unit
KW	Kruskal–Wallis test

## Appendix A

**Table A1:** Classification of participant diagnoses into major clinical categories.

Category	Diagnosis (Number of Participants)
Developmental disorders	Global developmental delay (3), DCD(1)
Neurological disorders	Cerebral palsy (11), Brain malformation (1), SDH (1), Spontaneous ICH (3), Acute necrotizing encephalopathy (1), Guillain–Barré syndrome (2)
Neuromuscular disorders	Duchenne muscular dystrophy (2), other myopathy (1), peripheral neuropathy (2), Spinal cord injury (1), Achilles tendinitis (1)
Genetic & chromosomal disorders	Down syndrome (1), Turner syndrome (1), Fragile X-syndrome (1), Pierre Robin sequence (1), Arthrogryposis multiplex congenita (1), 12p duplication (1), 10q11.22q11.23 duplication (1)
Orthopedic/foot alignment conditions	planovalgus deformity (2), scoliosis (1)

DCD, developmental coordination disorder; SDH, subdural hemorrhage; ICH, intracerebral hemorrhage.
